# Genome-wide identification, classification and expression analysis of the heat shock transcription factor family in Garlic (*Allium sativum* L.)

**DOI:** 10.1186/s12870-024-05018-3

**Published:** 2024-05-18

**Authors:** Xiaomeng Hao, Shutao He

**Affiliations:** 1 Institute of Neurobiology, Jining Medical University, Jining, China; 2 Institute of Biotechnology and Health, Beijing Academy of Science and Technology, Beijing, China

**Keywords:** Genome-wide, Heat shock transcription factors (HSFs), Garlic, Expression profile

## Abstract

**Background:**

The heat shock transcription factor (HSF) plays a crucial role in the regulatory network by coordinating responses to heat stress as well as other stress signaling pathways. Despite extensive studies on HSF functions in various plant species, our understanding of this gene family in garlic, an important crop with nutritional and medicinal value, remains limited. In this study, we conducted a comprehensive investigation of the entire garlic genome to elucidate the characteristics of the *AsHSF* gene family.

**Results:**

In this study, we identified a total of 17 *AsHSF* transcription factors. Phylogenetic analysis classified these transcription factors into three subfamilies: Class A (9 members), Class B (6 members), and Class C (2 members). Each subfamily was characterized by shared gene structures and conserved motifs. The evolutionary features of the *AsHSF* genes were investigated through a comprehensive analysis of chromosome location, conserved protein motifs, and gene duplication events. These findings suggested that the evolution of *AsHSF* genes is likely driven by both tandem and segmental duplication events. Moreover, the nucleotide diversity of the *AsHSF* genes decreased by only 0.0002% from wild garlic to local garlic, indicating a slight genetic bottleneck experienced by this gene family during domestication. Furthermore, the analysis of *cis*-acting elements in the promoters of *AsHSF* genes indicated their crucial roles in plant growth, development, and stress responses. qRT-PCR analysis, co-expression analysis, and protein interaction prediction collectively highlighted the significance of *Asa6G04911*. Subsequent experimental investigations using yeast two-hybridization and yeast induction experiments confirmed its interaction with HSP70/90, reinforcing its significance in heat stress.

**Conclusions:**

This study is the first to unravel and analyze the *AsHSF* genes in garlic, thereby opening up new avenues for understanding their functions. The insights gained from this research provide a valuable resource for future investigations, particularly in the functional analysis of *AsHSF* genes.

**Supplementary Information:**

The online version contains supplementary material available at 10.1186/s12870-024-05018-3.

## Background

Plant growth and development depend significantly on a timely and efficient response to biotic and abiotic stresses. Transcription factors are essential in regulating the expression of stress-responsive genes, which play vital roles in plant responses to stresses [1–3]. Heat shock transcription factors (HSFs) play essential roles in the response to heat stress and other stresses such as chilling, salinity, drought, and heavy metal toxicity [4–7]. By binding to heat stress elements (HSEs) in the promoters of heat stress-responsive genes, HSFs regulate the expression of heat shock proteins (HSPs), which function as molecular chaperones to prevent protein denaturation [8, 9]. Additionally, HSPs regulate protein folding, accumulation, localization, and degradation, and are believed to play significant roles in coping with various environmental stresses [10]. Furthermore, HSFs can regulate reactive oxygen species (ROS)-scavenging enzymes, such as ascorbate peroxidase (APX) and catalase (CAT) [11]. Therefore, HSFs play crucial roles in elucidating the molecular mechanisms underlying plant responses and adaptations to stresses. Besides, HSFs also participate in plant growth and development. Further studies on HSFs are necessary to develop strategies for crop improvement, addressing the challenges posed by global environmental changes [12–14].

HSFs, like other transcription factors, consist of multiple structurally and functionally conserved regions. A typical HSF protein possesses a DNA-binding domain (DBD) near the N-terminus, an adjacent oligomerization domain (OD) consisting of heptad repeats of hydrophobic amino acid residues (HR-A/B) and specific motifs such as nuclear localization signal (NLS), nuclear export signal (NES), activator motif (AHA motif) and repressor domain [8, 15]. DBD is the most conserved ingredient of HSFs, comprising three helical bundles (α1, α2, and α3) and four antiparallel β sheets (β1, β2, β3, β4) [16]. Besides, DBD domain provides HSF proteins with the specific ability to recognize the HSEs (HSEs: 50-AGAAnnTTCT-30) which are conserved palindromic binding motifs present in the promoter of heat stress-inducible genes [15, 17, 18]. Additionally, the HR-A/B regions have the characteristic of predicted coiled-coil structure, which is vital for the formation of active trimers [19]. Under various stress conditions, HSF proteins can assemble into active trimers to activate the expression of target genes, such as *HSP30*, *70* and *90*, by binding to *cis*-elements of these gene promoters [20–23]. Moreover, based on the variations in their HR-A/B domain, plant HSFs are categorized into three subfamilies: class A (subclasses A1, A2, A3, A4, A5, A6, A7, A8, and A9), class B (subclasses B1, B2, B3, and B4), and class C (subclasses C1 and C2) [24–26]. There are 21 or 7 amino acid residues inserted into HR-A/B region of Class A and Class C HSFs, respectively. However, class B HSFs have a heptad repeat pattern without insertion. Furthermore, the transport of HSFs into the nucleus can be assisted by NLS, and the NES regulates the distribution of HSFs between cytosol and nucleus. The AHA motif plays a crucial role as transcription activator in the class A HSFs, which is characterized by the presence of aromatic, large hydrophobic and acidic amino acid residues [27].

To date, the function of many members of HSF family have been analyzed in several plants including maize, rice, Chinese cabbage, wheat, carrot, soybean, Arabidopsis, cotton, legumes, poplar and barrel clover [28–37]. Experiments have confirmed that HSFs not only take part in resistance to heat but also are involved in response to abiotic or biotic stresses [38, 39]. For example, HSFA1a, HSFA1b, HSFA1d, HSFA1e and HSFA2 can act as the master regulators in the response to heat stress [40, 41]. As a typical representative of plant, *AtHSFA2* not only confers heat and osmotic stress tolerance, but also is essential for plant growth and development [42–44].The transcriptional activation activity of *AtHSFA6a* significantly increased under salt stress [45]. Overexpression of *GmHSFA1* was proved to improve heat stress tolerance of soybean [46]. The overexpression of *HSFA4a* can enhance Cd tolerance of wheat and rice [47]. By contrast, HSFBs are shown to be transcriptional repressors, for instance, *AtHSFB1* and *AtHSFB2b* negatively regulate heat responsive genes [48]. *OsHSFB2b* overexpression significantly reduced plant drought and salt tolerance [49]. Moreover, *OsHSFC1b* expression was strongly unregulated by salt, mannitol and ABA, not by H_2_O_2_, and *OsHSFC1b* overexpression improved salt and osmotic tolerance [50]. Moreover, *HSFA3* is necessary for the response to heat and drought stresses in Arabidopsis, but not in tomato [51], indicating that some HSFs have distinct functions in different species.

Garlic (*Allium sativum* L.), a perennial bulbous plant, is one of the most important economically vegetable, spice, and medicinal crops [51]. It originated from Central Asia 6000 years ago and had been cultivated for more than 5000 years all over the world [52]. Garlic is a diploid species (2n = 16), but the garlic genome is very large (16.9G) and complex, with high repetition rate (∼ 1.68%) and high heterozygosity of 80% [53]. The successful assembly of garlic chromosome-level genome provides basis for the comprehensive identification and characterization of the garlic HSFs families, which is crucial for exploring the molecular mechanism of garlic resistance to stresses. Here, we performed a comprehensive analysis of HSFs in garlic including physicochemical properties, phylogenetic relationships, motif distribution, intron-exon pattern, gene duplication events, expression profiles and preliminary functions. In addition, genomic variation, genetic diversity, and principal component analysis (PCA) were also analyzed. Our results provide valuable information for further studies of the biological functions of garlic HSFs.

## Results

### Identification and characterization analysis of HSFs in garlic

Totally, twenty-two genes encoding for HSFs proteins were identified in the whole genome using a range of HMM-based bioinformatics approaches. Among these genes, five genes (*Asa1G01216*, *Asa7G01431*, *Asa7G07097*, *Asa6G06026*, and *Asa2G05473*) were excluded due to the preservation of the DBD domain, coiled-coil structure, and gene integrity. Finally, 17 *AsHSF* genes were used for further analysis. The selected genes were ranked according to their physical locations (Table S1). The chromosome distribution displayed that the *AsHSF* genes were unevenly distributed on the chromosomes 1, 4, 5, 6, 7 and 8. Besides, *Asa0G01036*, *Asa0G02991* and *Asa0G03146* were discovered on unplaced scaffold. Chromosome 4 had the most abundant *AsHSF* genes with four, followed by chromosomes 6 and 8 with three genes. However, some chromosomes did not have the *AsHSF* genes such as chromosomes 2 and 3.

The characteristic analysis of AsHSFs revealed that Asa7G06422 was the shortest protein with a length of 176 amino acids, whereas the longest one was Asa5G00955 with a length of 477 amino acids (Table S1). The molecular weight (MW) of these proteins ranged from 20.57 (Asa7G06422) to 53.23 (Asa5G00955) kDa and their computed theoretical isoelectric points (pI) ranged from 4.67 (Asa5G00955) to 9.41 (Asa7G06422). The grand average of hydropathicity (GRAVY) value, which is correlated with the protein hydrophilicity, ranged from − 0.845 (Asa4G01727) and − 0.515 (Asa0G01036) for AsHSFs, suggesting that all AsHSFs are hydrophilic. The result of instability index revealed that all the other proteins, except for Asa4G02336 and Asa8G01031, were found to be unstable. Additionally, all AsHSFs were predicted to localize in the nucleaus. These findings indicate variations in the physical and chemical properties of AsHSF proteins, potentially attributed to the dissimilarities in non-conserved regions.

### Phylogenetic relationship, gene structure and conserved motif analysis

**Fig. 1:**
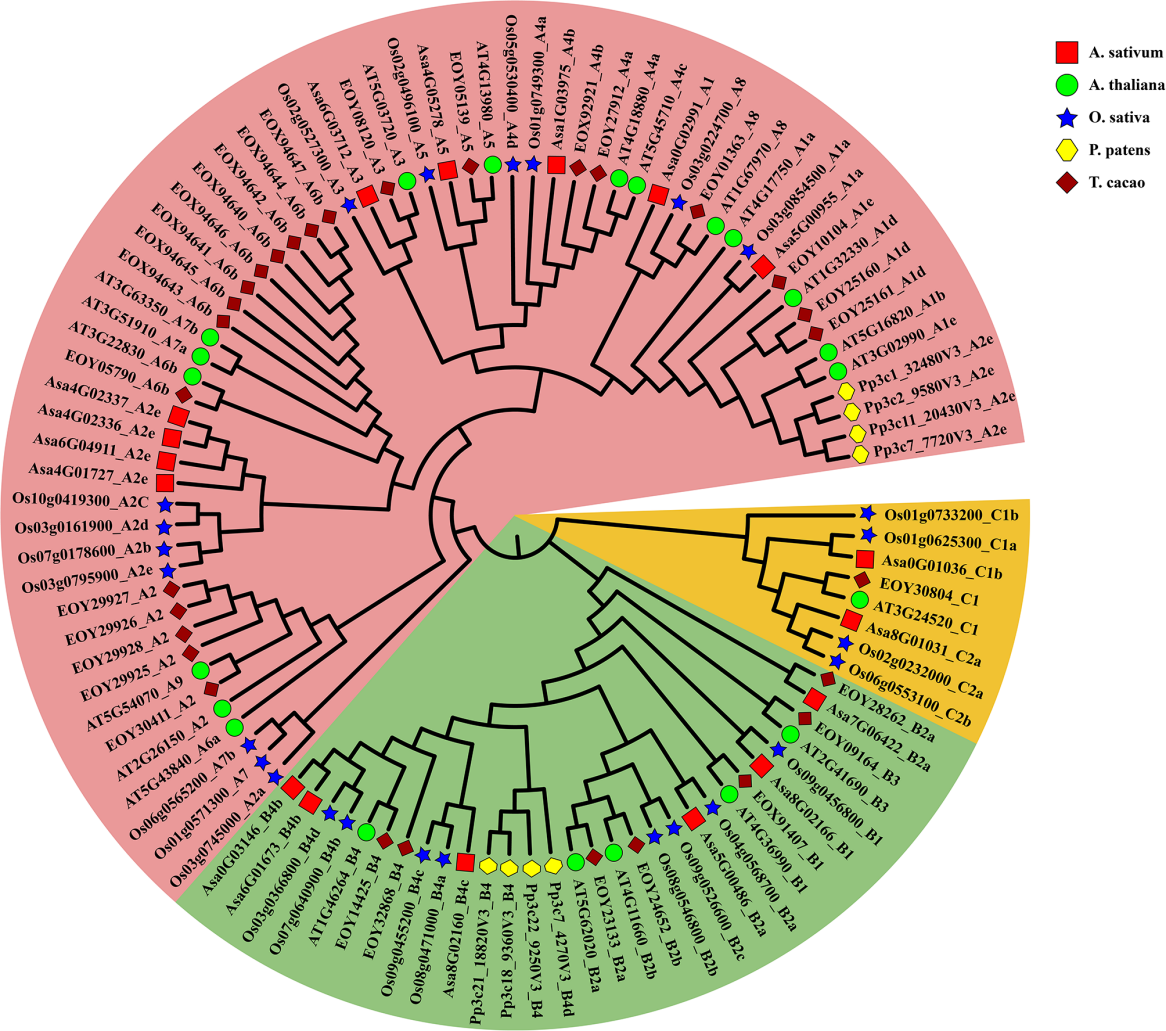
Phylogenetic analysis of HSF family proteins from *A. sativum* (Asa), *A. thaliana* (AT), *O. sativa* (Os), *P. patens* (Pp) and *T. cacao* (EOY). The phylogenetic tree was constructed using the maximum likelihood (ML) with 1000 bootstrap replicates. Classes A, B and C are filled in pink, light green and orange, respectively. Red solid squares, green solid circles, blue solid stars, yellow solid hexagons and brown solid diamonds represent proteins from *A. sativum*, *A. thaliana*, *O. sativa*, *P. patens*, and *T. cacao*, respectively

To investigate the phylogenetic relationships of HSF subfamilies among different species, we used the amino acid sequences of 17 proteins from *A. sativum*, 21 from *A. thaliana*, 25 from *O. sativa*, 8 from *P. patens*, and 30 from *T. cacao* to construct a phylogenetic tree using the maximum likelihood method (Fig. 1). The result showed that HSF proteins can be divided into three major subfamilies: classes A (pink), B (light green) and C (orange). The class A consisted of nine smaller clusters (A1-A9), representing the maximum of subclasses. Additionally, the class A subfamily had the highest number of HSF proteins, with 63 members across the five species, while the number of class C subfamily members was the smallest, only 8 members. The above result showed that the AsHSFs contained members of three subfamilies: 9 HSFAs, 6 HSFBs and 2 HSFCs.

**Fig. 2:**
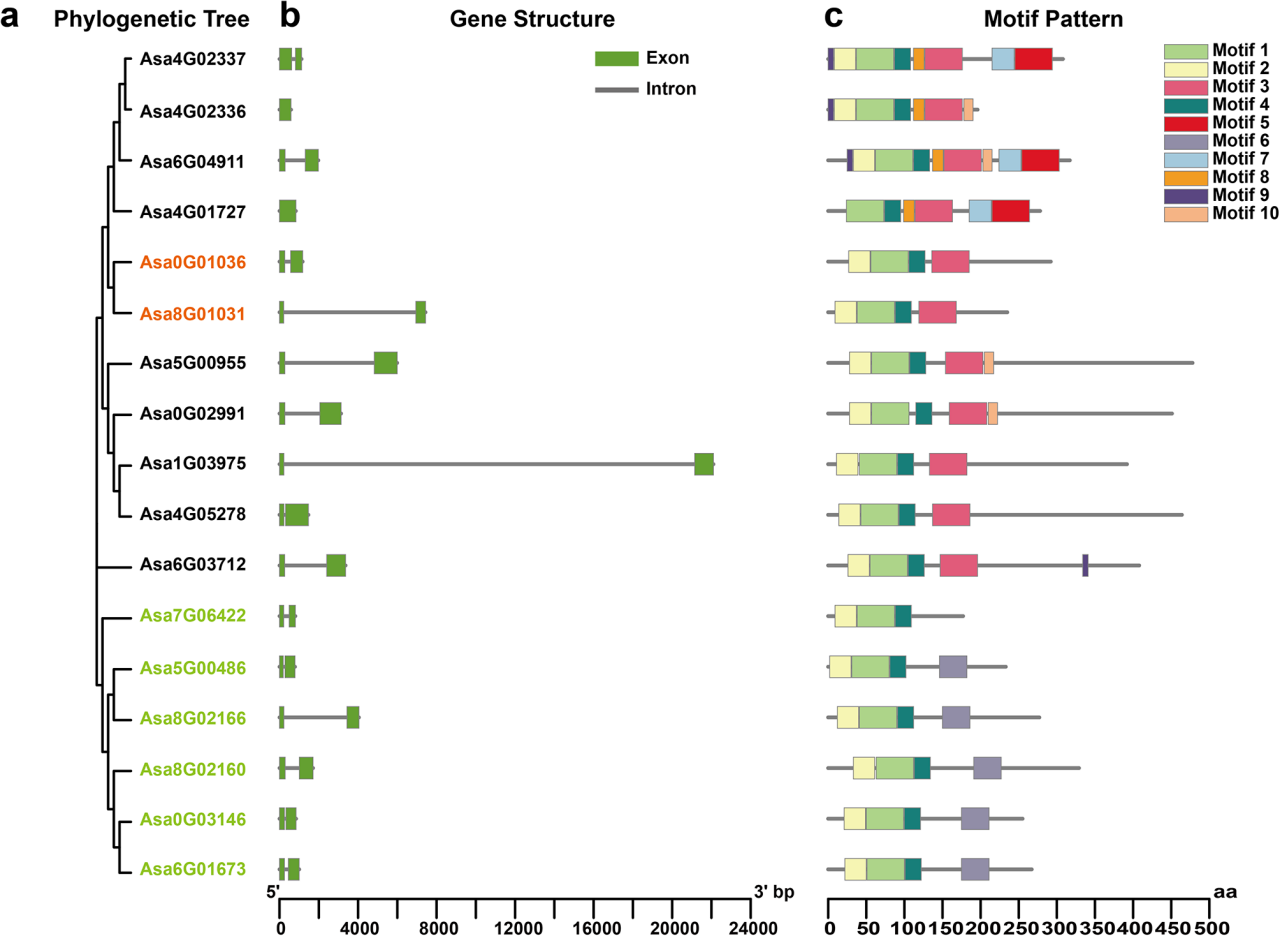
Architecture of phylogenetic tree, gene structure and conserved motifs of garlic HSFs. (**a**) Phylogenetic relationships of AsHSF proteins. Classes A, B and C are marked in black, light green and orange, respectively. (**b**) Exon-intron structures of garlic *HSF* genes. Green boxes and gray lines represent exons and introns, respectively. (**c**) The conserved motifs of AsHSF proteins. A total of ten motifs with the number 1–10 were identified and depicted in different colors. The sequence information of each motif is shown in Table S2. The protein length is indicated at the bottom of the figure

To further understand the structural variation of the AsHSFs, the intron and exon structures of *AsHSF* genes were investigated (Fig. 2a and b). The analysis showed that, except for *Asa4G02336* and *Asa4G01727*, other *AsHSFs* contained two exons. Besides, to analyze the protein sequence features of AsHSFs, MEME was used to search for the conserved motifs of AsHSF proteins. As a result, ten motifs with lengths ranging from 8 to 50 amino acids were identified (Fig. 2a and c, Table S2). The distribution patterns of the motifs (motif 1, 2, 4 and 6) were similar for class B, except for Asa7G06422, which lacked motif 6. Besides, the same motifs (motif 1, 2, 3 and 4) were detected in class C. Motifs 1 and 4 were detected in all AsHSFs, and motif 6 was only present in the members of AsHSFBs. The number of conserved motifs in Asa6G04911 was highest (9), and Asa7G06422 had the fewest (3).

Furthermore, the conserved domains in AsHSFs were analyzed (Supplementary Fig. 1, Table S3). The length of DBD domains and HR-A/B domains varied, with Asa0G02991 having the longest DBD domain (101 aa), Asa4G01727 having the smallest (87 aa), and most of DBD domains were 93 aa. Except for the DBD domain and HR-A/B domain, the other domains, like NLS domain, NES domain and AHA domain, were observed in AsHSF members. The majority of AsHSFs contained NLS domain whereas only four of seventeen AsHSFs possessed NES domain. In contrast, AHA motifs were present in the center of the C-terminal activation domains for the majority of class A members, while they were not detected in class B and C. Overall, despite variations in size and sequence, the members within a specific subfamily shared similar profiles of gene structure, conserved motif and domain, which confirmed the reliability of the phylogenetic tree.

### Chromosome localization, gene duplication and synteny analysis of *AsHSF* genes

**Fig. 3:**
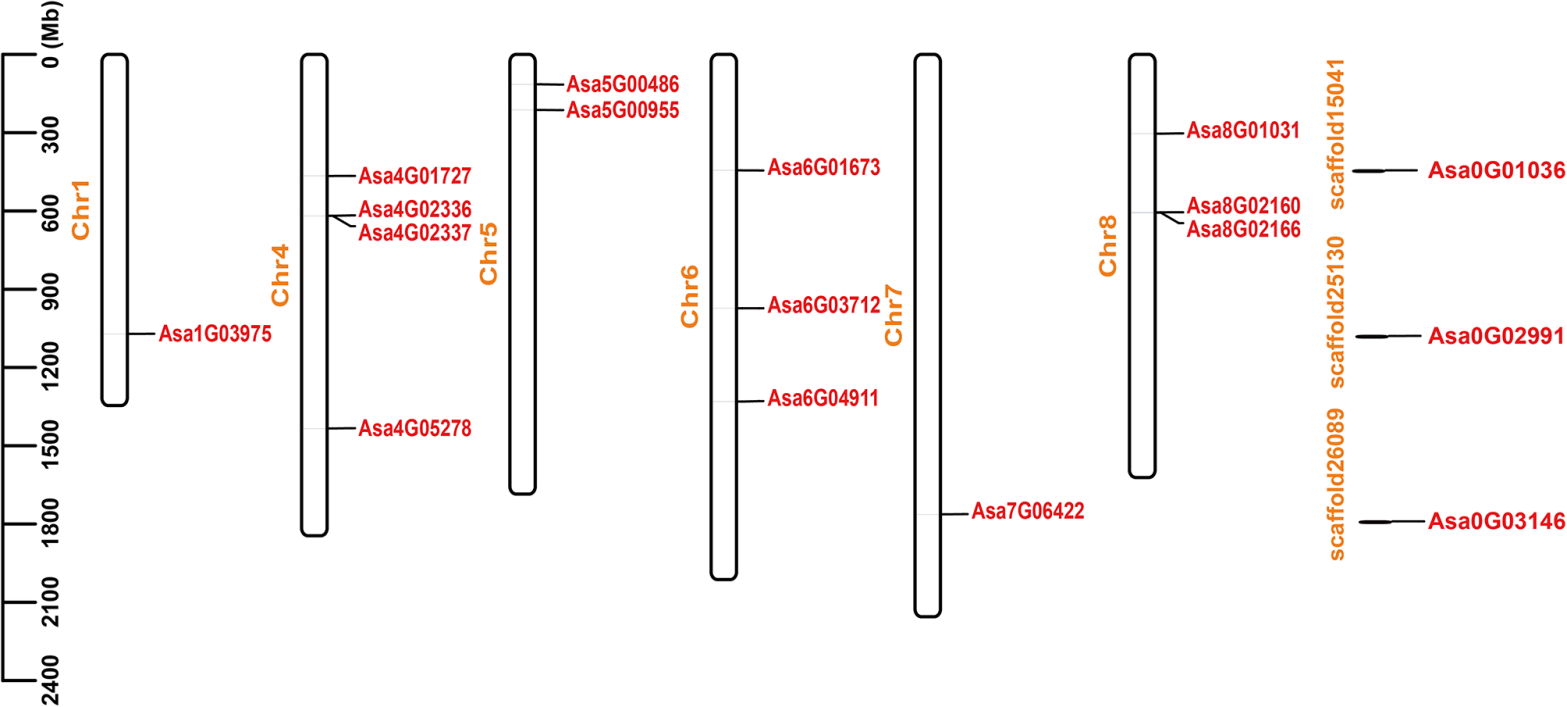
Chromosomes distribution of *AsHSF* genes. Individual chromosomes are represented by white bars, and the chromosome numbers are marked on the left side of the bars. The scale on the left is in million bases (Mb)

The analysis of chromosomal localization revealed the uneven arrangement of 14 *AsHSF* genes on 6 garlic chromosomes, and 3 *AsHSF* genes (*Asa0G01036*, *Asa0G02991* and *Asa0G03146*) were present on 3 scaffolds, respectively (Fig. 3, Table S1). There were 4 *AsHSF* genes localized on chromosome 4, ranking as the most abundant chromosome, whereas no genes were detected on chromosomes 2 and 3. The other 5 chromosomes contained 3 (chromosomes 6 and 8) to 1 (chromosomes 1 and 7) *AsHSF* genes. Additionally, no significant correlation between the chromosome length and the number of *AsHSF* genes was observed (Pearson correlation *r* = 0.3408, *p*-value = 0.4088), implying that longer chromosomes do not inherently harbor a greater number of *AsHSF* genes.

**Fig. 4:**
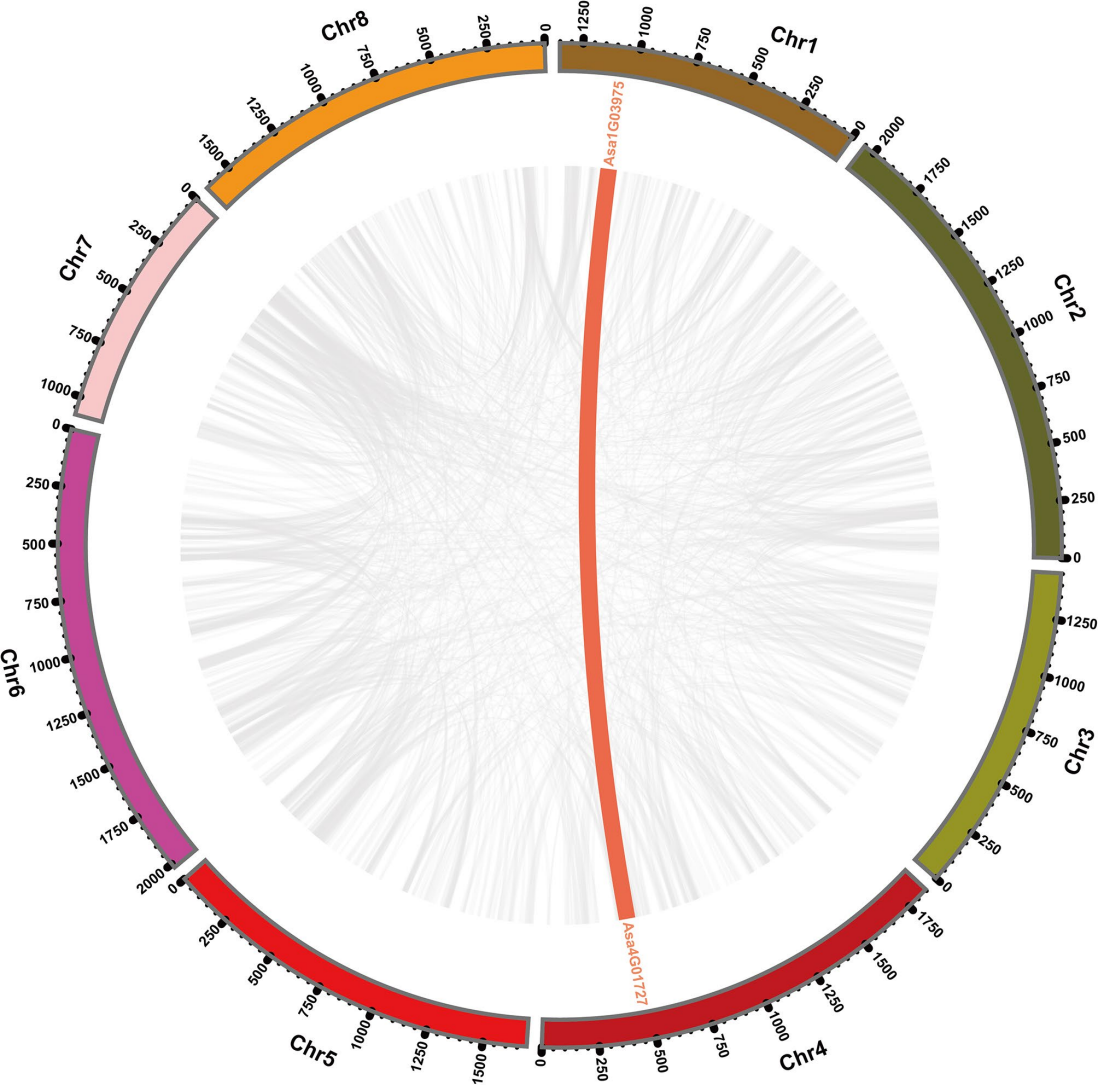
Interchromosomal relationships of *AsHSF* genes. Gray lines represent all syntenic relationships within the garlic genome, while collinear blocks of *AsHSF* genes are indicated by red lines

To investigate the gene expansion mechanism of *AsHSFs*, gene duplication event analysis was conducted. Two tandem duplicated regions including 4 *AsHSF* genes (*Asa4G02336* and *Asa4G02337*, *Asa8G02160* and *Asa8G02166*) were identified on chromosomes 4 and 8, implying hot spots of *AsHSF* gene distribution (Fig. 3, Table S4). Additionally, only one pair of segmentally duplicated genes (*Asa1G03975* and *Asa4G01727*) was identified, which were related with chromosomes 1 and 4 (Fig. 4, Table S4). The above results indicated that both tandem and segmental duplication might be the key evolutionary driving force of *AsHSF* genes.

To evaluate the evolutionary restrictions of *AsHSF* genes, the non-synonymous (Ka) vs. synonymous (Ks) substitution ratios of the above duplicated gene pairs were estimated. Interestingly, the Ka/Ks ratio between *Asa4G02336* and *Asa4G02337* was greater than 1, implying positive selection, whereas that between *Asa4G01727* and *Asa1G03975* was less than 1, indicating purifying selection (Table S4). Additionally, the Ka/Ks ratio between *Asa8G02160* and *Asa8G02166* was almost equal to 1, indicating neutral selection during gene expansion process.

### Evolutionary analysis of *HSF* genes in garlic and several different species

**Fig. 5:**
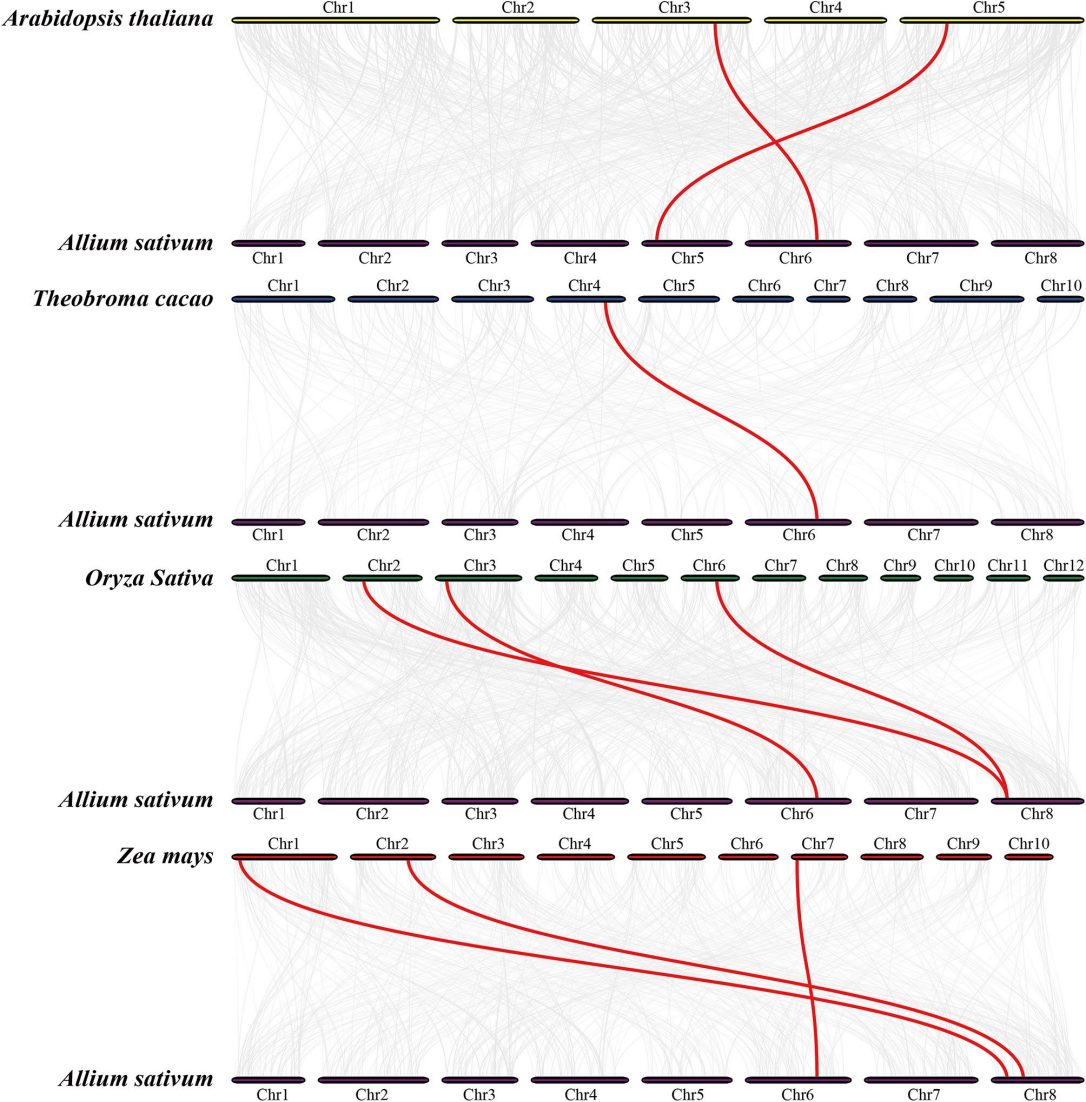
Syntenic analysis of *HSF* genes between garlic and four representative plant species. Grey lines represent all collinear blocks between the paired genomes, while red lines highlight syntenic *HSF* gene pairs

To further analyze the evolutionary mechanisms of the *AsHSF* genes family, syntenic relationships with four representative plants, including two monocotyledonous plants (*Oryza sativa* and *Zea mays*) and two dicotyledonous plants (*Arabidopsis thaliana* and *Theobroma cacao*) were constructed (Fig. 5). A total of 4 *HSF* genes in garlic had syntenic relationships with those in four plants, of which 3, 3, 2 and 1 syntenic gene pairs were detected with two monocots (*Zea mays* and *Oryza sativa*) and two dicots (*Arabidopsis thaliana* and *Theobroma cacao*), respectively. The result is consistent with the evolutionary associations between garlic and these plants, indicating that these evolutionarily conserved genes may play vital roles during evolution. Additionally, the Ka/Ks ratios between garlic and the monocots had no significant difference with those between garlic and the dicots (Table S5).

### Nucleotide variation and population structure of *AsHSF* genes

Garlic resequencing data was used to investigate *AsHSF*-associated single nucleotide polymorphisms (SNPs) which can reflect the sequence diversity of *AsHSF* genes. The SNP call pipeline identified 918 high confidence SNPs (Table S6). Most of SNPs associated with *AsHSFs* were enriched in the intergenic region, while others were present with in the genic regions, involving 1 missense, 5 intron, 7 downstream, and 4 upstream variants. Besides, the overall transition/transversion (Ts/Tv) ratio was 1.981, with C/T (21.02%) and G/A (21.46%) being the most prevalent allelic substitution profiles. These results implied a higher occurrence of purine to purine or pyrimidine to pyrimidine mutations compared to pyrimidine to purine or purine to pyrimidine mutations.

**Fig. 6:**
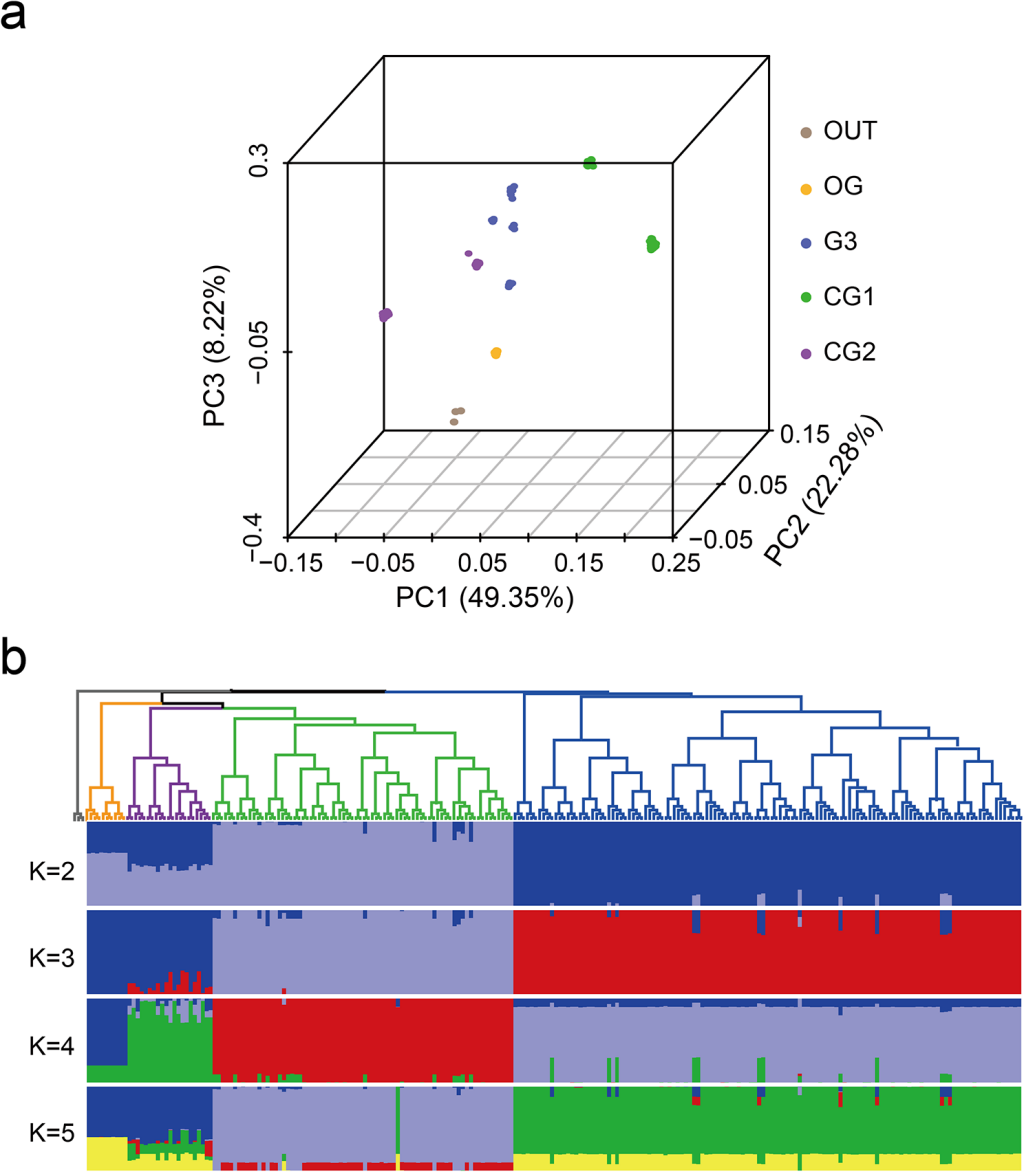
Population structure of wild garlic and local garlic according to *AsHSF*-related SNPs. (**a**) Principal component analysis plots of the first component (PC1), second component (PC2) and third component (PC3). The color of dots represents the population and location. (**b**) Phylogenetic tree and population structure with K ranging from 2 to 5. The rooted tree was constructed using neighbor-joining method. The orders and positions of sample accessions on the x-axis are consistent with those in the neighbor-joining tree

To further investigate the relatedness between the origin group and three different garlic cultivars, PCA using *AsHSF*-related SNPs was carried out (Fig. 6a). The first eigenvector, which explained 49.35% of the genetic variance, revealed divergence among these populations. The second and third eigenvector were used to distinguish wild-type and endemic garlic, accounting for 22.28% and 8.22% of the genetic variation, respectively. Similar population relationships were observed in the phylogenetic tree (Fig. 6b). ADMIXTURE analysis further confirmed the consistent group relatedness observed in the phylogenetic tree, exhibiting exact joining relationships. When K = 4, noticeable biological differences between wild garlic and local garlic were observed, and with an increase to K = 5, there was a clear separation according to geographical origin. The presence of genetic mixing between wild and landraces garlic indicates the underlying domestication of cultivated garlic and ongoing gene flow between wild and landraces garlic.

Population-based nucleotide diversity was estimated to evaluate the emergence of previous genetic bottlenecks of *AsHSF* genes during garlic acclimation. The nucleotide diversity of *AsHSF* genes from wild garlic to local garlic decreased by only 0.0002% (Supplementary Fig. 2), suggesting a mild genetic bottleneck experienced by this gene family during domestication. Besides, Wright’s F-statistic (Fst) was used to assess population differentiation. The Fst index between wild and landrace garlic within the *AsHSFs* was 0.2121, suggesting that *AsHSFs* did not suffer from strong selective pressure during garlic domestication.

### *Cis*-acting elements of garlic *AsHSF* genes

**Fig. 7:**
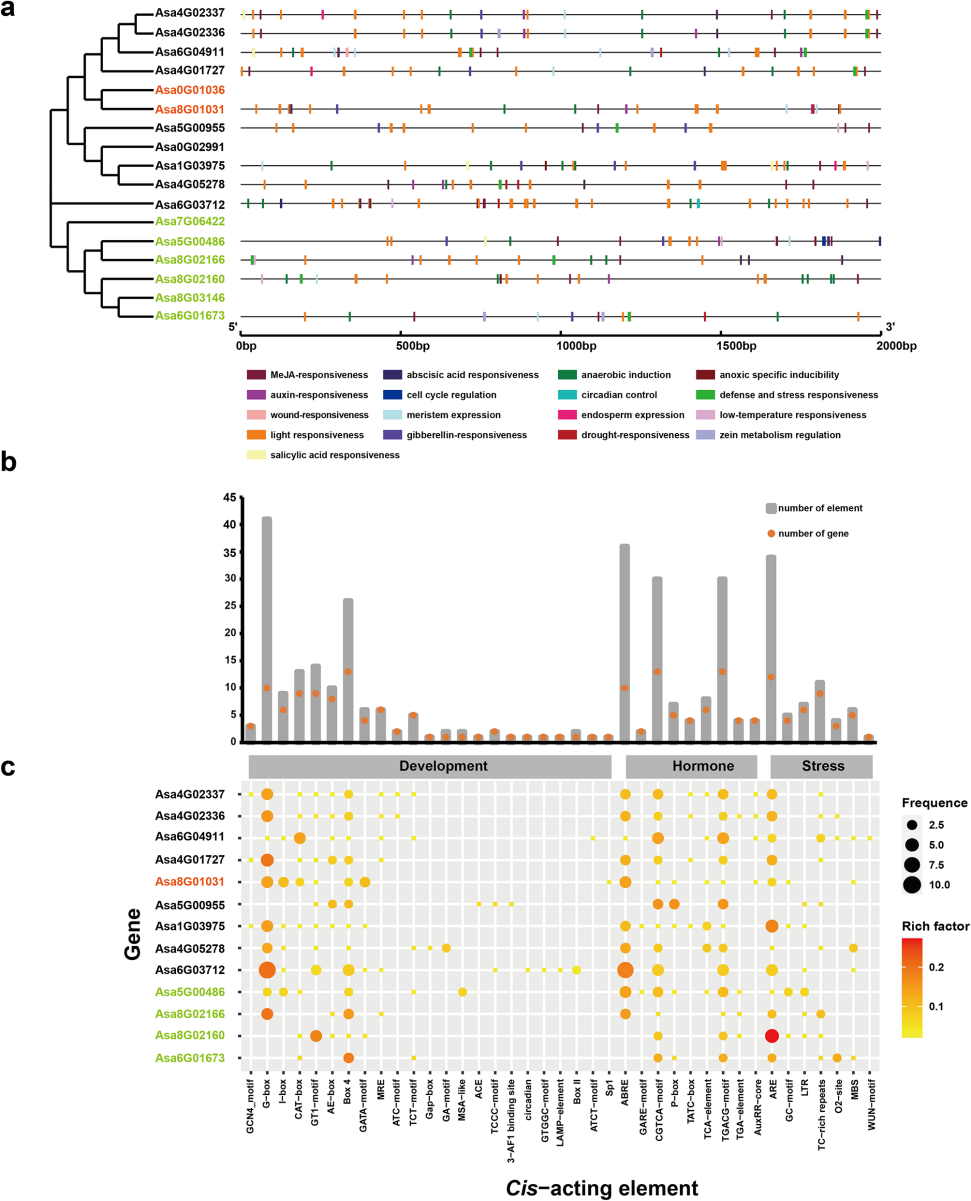
*Cis*-acting elements in the promoter of the *AsHSF* genes. (**a**) Diagram illustrating the present of *cis*-acting elements in the promoter of the *AsHSF* genes. Each *cis*-acting elements is indicated by a rectangular box of a specific color. (**b**) Number of each *cis*-regulatory element in the promoter regions of the *AsHSF* genes. Red dots represent the number of genes containing the corresponding *cis*-acting element, while the total number of *cis*-acting elements is represented by gray boxes. (**c**) Frequency of each *cis*-regulatory element in the *AsHSF* gene promoters. The *cis*-acting elements are categorized into development-related, hormone-related and stress-related classes according to the feature notes

To predict the possible biological functions of the *HSF* family in garlic, *cis*-acting elements in the promoter of these genes were investigated. Owing to the limited assembly integrity of garlic genome, the promoter sequences of 4 genes (*Asa0G01036*, *Asa0G02991*, *Asa7G06422* and *Asa8G03146*) were not retrieved. Therefore, we further investigated the *cis*-acting elements of 13 *AsHSF* genes (Fig. 7a). The result revealed that these predicted *cis*-acting elements can be divided into three classifications (developmental, hormone and stress process-related elements) (Fig. 7b and c). Among these, developmental-related elements were the most abundant, including the CAT-box associated with meristem expression, which were present in the promoter regions of most *AsHSF* genes. Furthermore, ARE element (essential for anaerobic induction), LTR element (related to low-temperature) and MBS (related to drought-inducibility) were detected in the promoters of 12, 6 and 5 *AsHSF* genes, respectively. All *AsHSF* gene promoters possessed the CGTCA-motif and TGACG motif (associated with methyl jasmonate (MeJA) responsiveness). Furthermore, the ABA-responsive element (ABRE), salicylic acid responsiveness (TCA-element) and the auxin-responsiveness element (TGA-element and AuxRR-core) were found in 10, 6, 4 and 4 *AsHSFs*, respectively. The promoter regions of 2, 5 and 4 genes contained elements associated with gibberellin responsiveness, such as GARE-motif, P-box and TATC-box. These results showed that transcriptional regulation of *AsHSFs* is related to hormones, development and stress.

### Expression profile analysis of *AsHSF* genes under various abiotic stresses

**Fig. 8:**
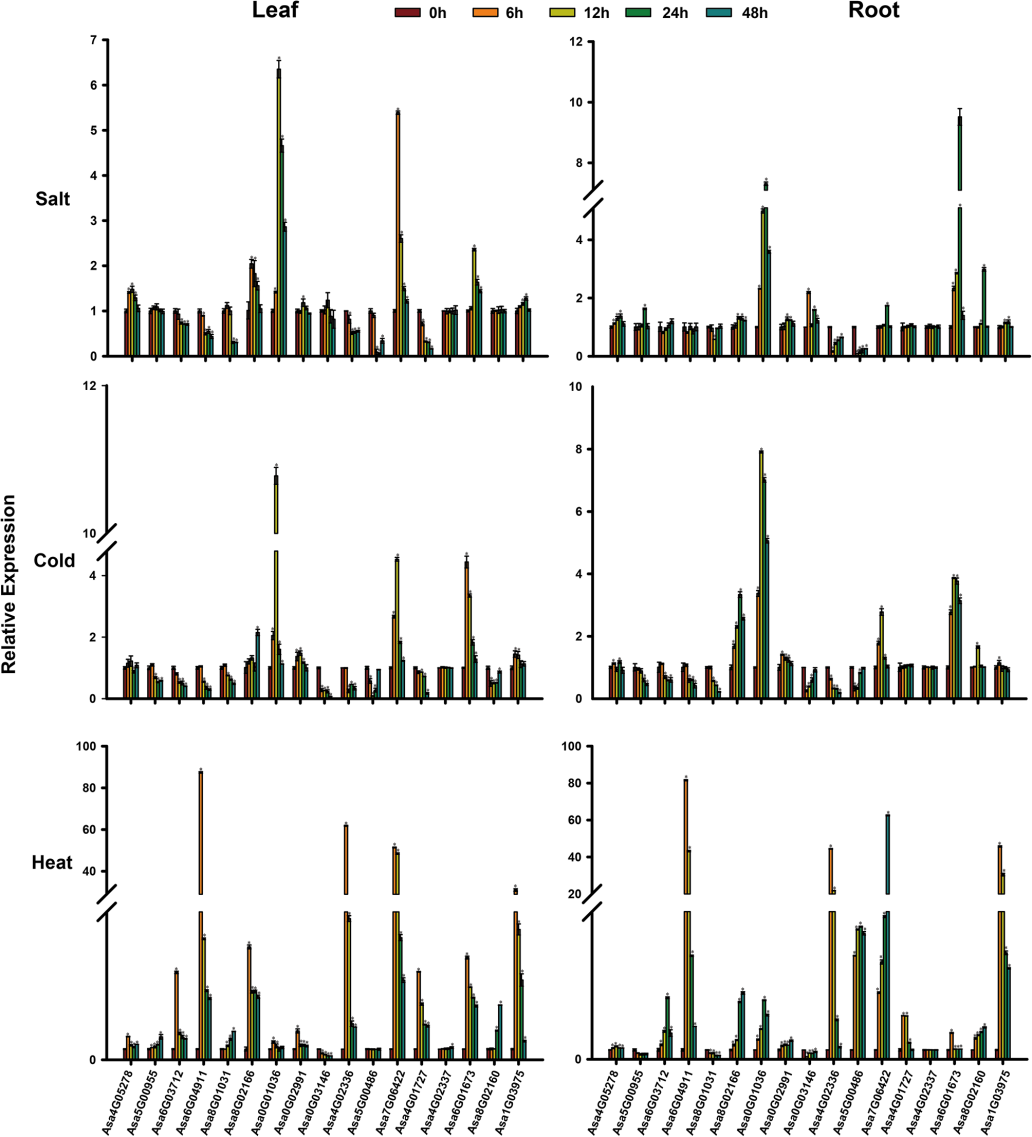
Expression analysis of *AsHSF* genes in response to salt, cold and heat using qRT-PCR. The expression data were normalized to 1 in unstressed plants (0 h). Error bars indicate standard deviations from the biological replicates. One asterisk (*) represents a significant difference at *P* < 0.05, determined by Student’s *t*-test

To further analyze the potential physiological function of *AsHSF* genes under various abiotic stress, such as salt, heat and cold treatment, the expression patterns of these genes were detected via qRT-PCR (Fig. 8). For salt stress, all 17 *AsHSF* genes were detected in leaves and roots, of which 7 upregulated genes and 6 downregulated genes were found in leaves, and 10 upregulated and 3 downregulated *AsHSF* genes were detected in roots. Among them, in both the root and leaf, seven genes (*Asa4G05278*, *Asa8G02166*, *Asa0G01036*, *Asa0G02991*, *Asa7G06422*, *Asa6G01673*, *Asa1G03975*) exhibited induced expression, while three genes (*Asa8G01031*, *Asa4G02336*, *Asa5G00486*) displayed inhibited expression. For cold stress, 6 upregulated *AsHSF* genes and 10 downregulated *AsHSF* genes were identified in leaves, and 8 upregulated *AsHSF* genes and 7 downregulated *AsHSF* genes were found in roots. In both the root and leaf, six genes (*Asa8G02166*, *Asa0G01036*, *Asa0G02991*, *Asa7G06422*, *Asa6G01673*, *Asa1G03975*) were upregulated, and *Asa0G01036* had the most significantly induced expression with the highest expression level at 12 h. In addition, *Asa5G00955*, *Asa6G03712*, *Asa6G04911*, *Asa8G01031*, *Asa0G03146*, *Asa4G02336* and *Asa5G00486* were inhibited in leaves and roots. For heat stress, the majority of genes were induced to express in both the root and leaf. Interestingly, *Asa6G04911* exhibited the highest induced expression level in both the leaf and root, suggesting its potential key role in responding to heat stress. Moreover, the expression level of *Asa6G03712*, *Asa6G04911*, *Asa8G01031*, *Asa4G02336* and *Asa4G01727* were up-regulated under heat stress, and down-regulated under cold and salt stresses in leaves. *Asa5G00955*, *Asa0G03146* and *Asa8G02160* responded to temperature stress but not salt stress in leaf, whereas *Asa6G03712* and *Asa6G04911* responded to temperature stress but not salt stress in root. The expression level of *Asa8G02166*, *Asa0G01036*, *Asa0G02991*, *Asa7G06422*, *Asa6G01673* and *Asa1G03975* were up-regulated in leaf and root under three stresses (heat, cold and salt). The distinct expression patterns of individual *AsHSF* suggested the diverse roles of each *AsHSF* gene under different biotic stresses.

### Gene co-expression analysis

**Fig. 9:**
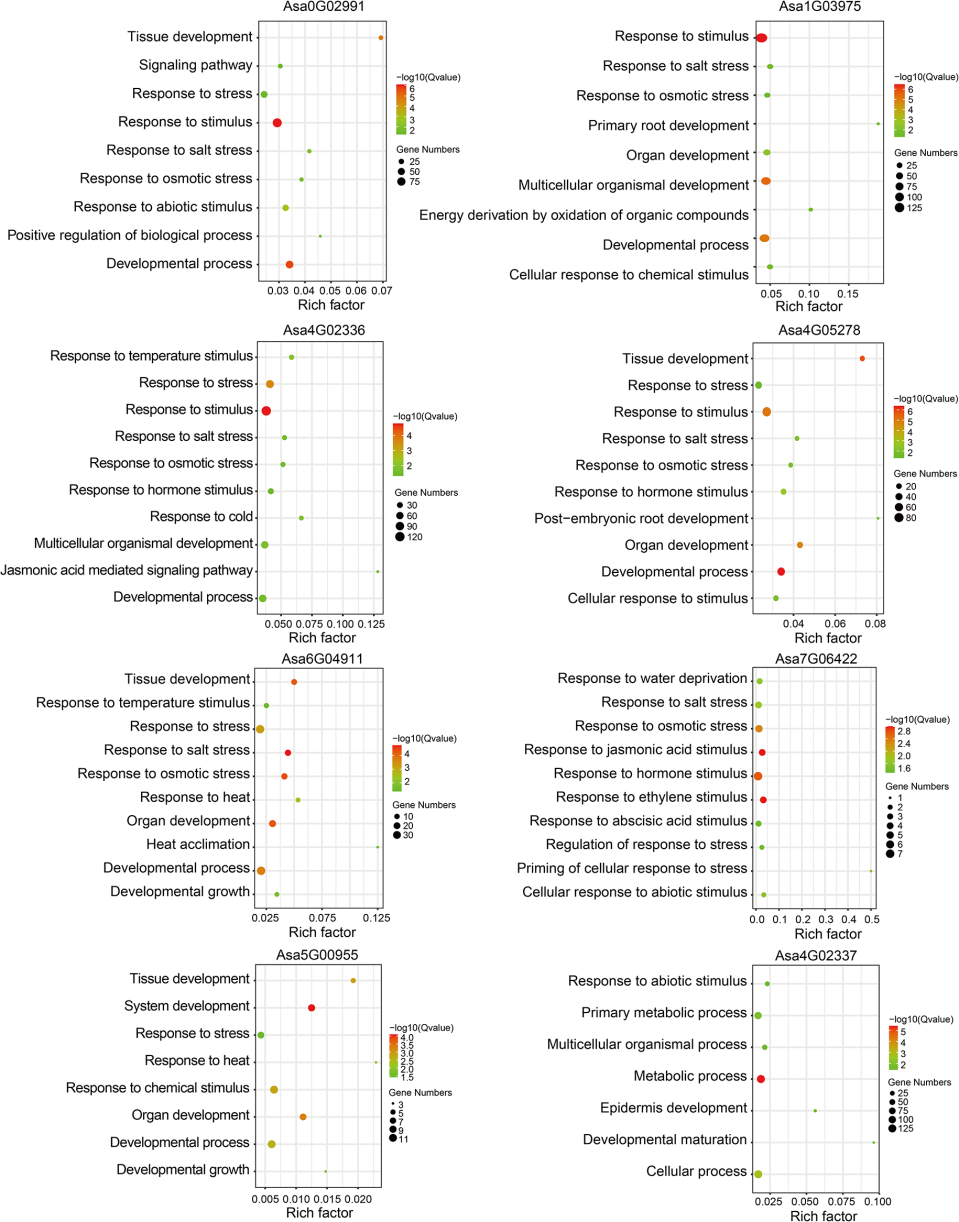
GO enrichment analysis of eight co-expressed gene sets. The size of each spot represents the number of genes enriched in specific term. The color of each spot indicates the enrichment significance level

Co-expression network analysis can effectively annotate gene function [54]. The co-expression network of *AsHSF* genes was constructed based on a massive dataset of 185 RNA-Seq samples by using Weighted Gene Correlation Network Analysis (Table S7). A total of 8 co-expression networks were acquired (Fig. 9). The largest network was centered on *Asa4G02336* (1156 genes), whereas the smallest network took *Asa5G00955* as the core (46 genes) (Supplementary Fig. 3).

To investigate the potential biological processes in which these genes may be involved, GO enrichment analysis was further investigated. All of *AsHSF* genes contained the GO terms associated with stress response, such as response to stress, response to abiotic stimulus and response to osmotic stress, indicating that *AsHSF* genes might be crucial for stress response (Fig. 9). Particularly, among these genes, *Asa6G04911* and *Asa5G00955* were enriched in GO terms related to heat response, including response to heat and heat acclimation, suggesting key regulatory roles of these genes in heat response process. Additionally, except for *Asa7G06422*, the co-expressed networks of most *AsHSF* genes were enriched in developmental process, tissue development and organ development, suggesting that these genes might be associated with plant growth and development. Moreover, several GO terms related to hormone response, such as response to hormone stimulus, jasmonic acid mediated signaling pathway, and response to ethylene stimulus, were enriched in the co-expressed network of several *AsHSF* genes (*Asa4G02336, Asa4G05278 and Asa7G06422*), implying the potential vital function of these genes in the regulation of hormone response.

### Protein interaction network of AsHSFs

To further predict the functions of AsHSFs, STRING was used to analyze the functional and physical protein associations of the AsHSF proteins (Supplementary Fig. 4). The results revealed that the interaction network contain 17 AsHSF proteins and 10 known Arabidopsis proteins. The results of GO enrichment analysis revealed that the majority of AsHSFs exhibited enrichment in various biological processes. Among them, the most highly enriched processes were cellular response to heat, positive regulation of response to heat stress and response to temperature stimulus (Supplementary Fig. 5).

### The functional analysis of *Asa6G04911*

**Fig. 10:**
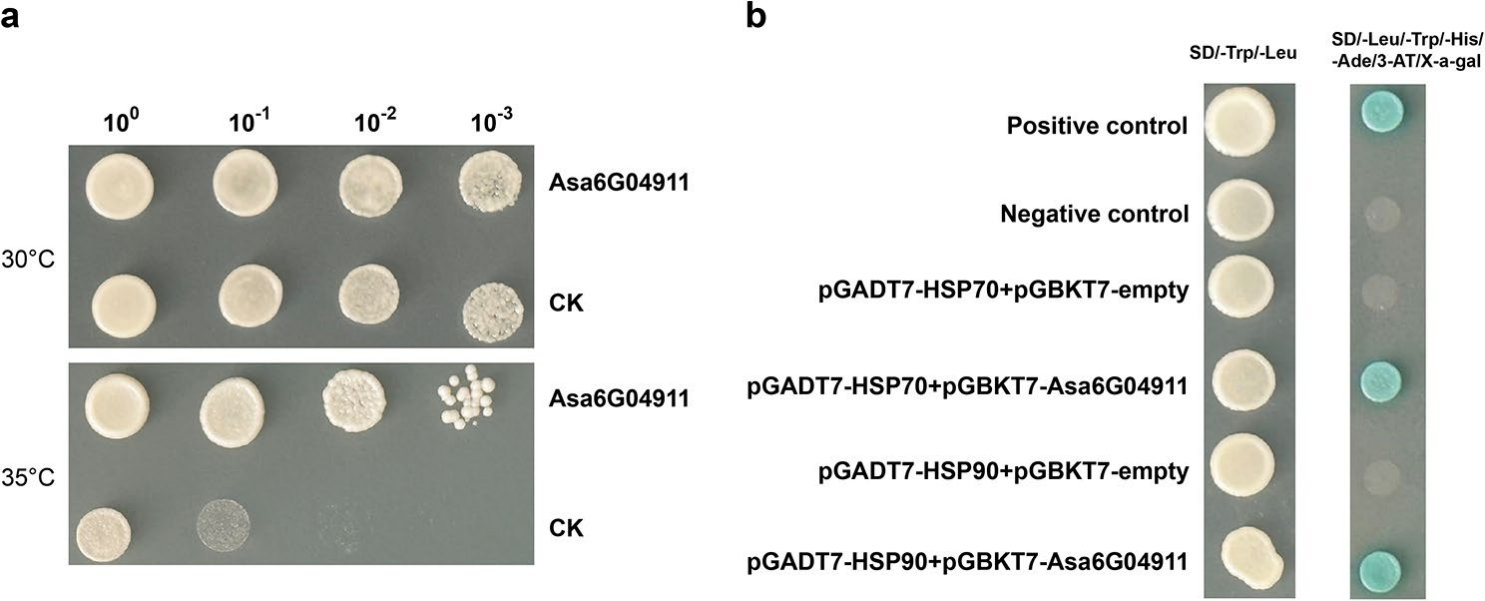
The functional analysis of *Asa6G04911*. (**a**) The control strain and the recombinant strain harboring *Asa6G04911* were incubated on SG/-Ura plates at 30 °C and 35 °C for 3 days, respectively. (**b**) Yeast two-hybrid assay of Asa6G04911 and HSP70/90. The positive control (53-pGBKT7 + T-pGADT7) and negative control (53-pGBKT7 + lam-pGADT7) were employed for comparison

The result of qRT-PCR analysis revealed that *Asa6G04911* exhibited the most significant alteration in response to heat stress, and GO enrichment of co-expression analysis showed that *Asa6G04911* was associated with heat-related processes. To gain deeper insights into the function of *Asa6G04911* in heat stress, we introduced this gene into the *S. cerevisiae* BY4741 strain to assess its performance under heat stress. At 30 °C, there were no significant growth differences between the control strain (BY4741 transformed with an empty vector) and the recombinant strain harboring *Asa6G04911*. However, at 35 °C, the recombinant strain displayed a significantly accelerated growth rate compared to the control strain (Fig. 10a). These findings strongly suggested the key function of *Asa6G04911* gene in the response to heat stress.

Additionally, it is predicted that Asa6G04911 might interact with HSP70 and HSP90 using STRING (Supplementary Fig. 4). To verify these interactions, yeast two-hybrid (Y2H) assay was conducted. As depicted in Fig. 10b, both yeast transformants (Asa6G04911-BD and HSP70-AD) and yeast transformants (Asa6G04911-BD and HSP90-AD) exhibited blue color on SD/-Leu/-Trp/-His/-Ade/3-AT/x-α-gal, which verified the specific interactions between Asa6G04911 and HSP70/90.

## Discussion

Global warming has caused serious abiotic stresses and an increasing number of reports support the crucial role of *HSF* genes in regulating the expression of genes related to biotic and abiotic stresses [34, 55]. Furthermore, HSF family members have been investigated and characterized in various plants, including maize [56], peach [57], wheat [58], potato [59], cotton [32], and Chinese cabbage [34]. However, a comprehensive study of *AsHSF* genes at the whole genome level in garlic has not been conducted so far. In this study, we conducted a comprehensive analysis of the *AsHSF* genes, resulting in the discovery of 17 full length *HSF* genes distributed across the garlic genome (Table S1). The number of *HSF* genes in garlic was lower than wheat (82) [60], rice (25) [61], tomato (26) [62], soybean (26) [28] and sorghum (25) [63]. Previous studies have demonstrated that different species harbor varying amounts of *HSF* transcription factors due to their distinct growth environments, and land plants generally possess a higher number of *HSF* genes compared to algae [36]. The diversification of HSFs in plants may be attributed to gene and genome-wide replication events, such as whole-genome duplication (WGD), occurring at distinct evolutionary stages, followed by gene loss [64]. Additionally, the result of theoretical isoelectric point implied that Asa4G02336, Asa7G06422, Asa8G02160 and Asa8G02166 might be basic protein, while the others were acidic proteins (Table S1), indicating their potential important roles in diverse microenvironments [65]. Furthermore, the GRAVY results showed that all the AsHSF proteins were hydrophilic, which is consistent with the findings in potato [59], Chinese cabbage [34] and carnation [66].

Phylogenetic analysis revealed that these identified garlic genes could be classified into three main classes, namely A, B and C, with 9, 6 and 2 members, respectively (Fig. 1). Similar to *Arabidopsis thaliana*, *Oryza sativa* and *Theobroma cacao*, class A was the most abundant subclass in garlic, surpassing class B and C in terms of member count. In comparison to Arabidopsis, subclass A6, A7, A8 and A9 were lost in the garlic’s HSF family, indicating that gene loss might have happened during the evolutionary process. Moreover, the modular structure of garlic HSF proteins was highly conserved, and the distribution of motifs showed that the majority of members within the same subclass exhibited similar protein motifs, providing additional insights into the evolutionary relationship of AsHSFs. Additionally, there are indications that gene intronic profiles are closely associated with gene function, and genes that require rapid activation in response to stress are inclined to evolve to gene with reduced intron density [67]. Although the intron number of most *AsHSF* genes were similar, their intron length varied, which is consistent with observations in potato [59] and carnation [66]. The differences in intron length and intron location of *AsHSF* genes indicate potential functional variations.

Various studies have reported that gene duplication, including tandem duplication and segmental duplication, might be the main driving force for the expansion of each family and the acquisition of novel gene functions [68]. Another report revealed that garlic has undergone three WGD events [53]. Here, only three pairs of *AsHSFs* were underwent gene duplication, including two pairs of tandem (*Asa4G02336* and *Asa4G02337*, *Asa8G02160* and *Asa8G02166*) and one segmental (*Asa1G03975* and *Asa4G01727*) duplication events, implying that gene duplication events might play vital roles in the gene expansion of *AsHSFs*. Additionally, the collinearity between garlic *HSFs* and those of two monocots and two dicots was analyzed. More collinearity gene pairs were observed between garlic and monocots, compared with those between garlic and dicots, which is consistent with the relative phylogenetic relationships among these species. The results of Ka/Ks ratios between these gene pairs suggested that these genes might undergo different evolutionary selection pressures.

When wild species are subjected to a new selection environment related to human demands, it will slowly lead to distinct morphological and physical characteristics alterations in the species, eventually, making it separated from its wild ancestors. This is a process of domestication which is co-evolution of plants and animals [69, 70]. Currently, there is limited knowledge about the alterations in *AsHSFs* caused by garlic domestication. The genetic variations in *AsHSF* genes between wild and domesticated garlic populations were analyzed using whole-genome resequencing data. A total of 918 *AsHSFs*-related SNPs were identified, and we observed an uneven distribution of SNPs in the genome sequence, which is consistent with previous studies [71]. PCA, admixture and phylogenetic analysis effectively separated all accessions into two groups: wild garlic and local garlic. In addition, the assessment of nucleotide diversity in the population revealed that the *AsHSF* genes encountered a slight genetic bottleneck during domestication, and the result of Fst indicated that the *AsHSF* genes did not undergo strong selection pressure during domestication.

Compared with animals, plants are constantly exposed to environmental stresses, therefore a series of molecular mechanisms to cope with changes in the external environment have evolved in plants. Promoter analysis revealed significant differences in the number and types of *cis*-acting elements among *AsHSF* genes, suggesting that *AsHSFs* may play diverse roles in different types of stresses (Fig. 7). However, it is interesting that no *cis*-acting elements associated with heat were present in the promoter of *AsHSF* genes, which is consistent with studies in potato [59] and carnation [66]. The precise regulatory mechanisms of *AsHSF* genes need to be further investigated. The expression patterns of *AsHSFs* under different stresses were studied by qRT-PCR. The majority of *AsHSFs* responded to temperature stress, indicating that *AsHSFs* might play very important roles in the process of temperature stress response, and the different expression patterns of each gene under diverse stresses suggest that *AsHSFs* might have distinct functions in various abiotic stress processes. Thus, these genes hold significant potential as candidate genes for the study of stress resistance in garlic. In addition, the results showed that the majority of genes could respond to heat stress. Particularly, *Asa6G04911* was the most significantly induced *AsHSF* gene in heat stress, suggesting that this gene might have specific functions for heat stress response and related signal transduction. Moreover, the GO enrichment result of co-expression analysis further predicted that *Asa6G04911* might have a vital role in the heat response process, which was validated by the yeast-induced expression experiment. In addition, protein interaction network showed that Asa6G04911 might interact with HSP70/90, which was further proved by yeast two-hybrid experiment. Our study laid a foundation for further investigation on the molecular functional mechanism of Asa6G04911 in garlic.

## Conclusions

In this study, we conducted a comprehensive analysis of the garlic *HSF* gene family at the genome-wide level. We successfully identified a total of 17 genes classified into 3 classes. Additionally, we conducted analyses on various aspects, including gene structure, motif composition, chromosome localization, gene duplication events, nucleotide variations, and population structure of the *AsHSF* genes, providing insights into the evolutionary characteristics of *AsHSF* genes. Moreover, we investigated the functional aspects of the *AsHSF* genes by examining promoter *cis*-regulatory elements, expression patterns, co-expression analysis, and protein interaction prediction. Notably, our findings confirmed that Asa6G04911 plays a crucial role in response to heat stress and can interact with HSP70/90. These findings improve our understanding of the roles of *AsHSF* genes in heat stress response and provide a good foundation for the further investigation of the molecular regulatory mechanisms underlying the regulation of heat stress in garlic.

## Methods

### Identification and sequence analysis of the HSF members in garlic

Whole genome sequence and annotation data of garlic were retrieved from 10.6084/m9.figshare.12570947.v1, and those of the other five species, namely *Arabidopsis thaliana*, *Oryza sativa*, *Physcomitrella patens, Zea mays* and *Theobroma cacao*, were retrieved from the Phytozome (https://phytozome-next.jgi.doe.gov/) and Ensembl Plants database (http://plants.ensembl.org/index.html). HMMER 3.0 was used to conduct the HMM search with a threshold of E < 1e^− 5^ to explore the HSF domain (PF00447) which was obtained from the PFAM database (http://pfam.xfam.org/). Moreover, BLASTP was utilized to search the garlic proteins with a threshold of 1e^− 5^ for the e-value and 50% for the identity, using the HSF protein sequences of rice and Arabidopsis retrieved from PlantTFDB (http://planttfdb.gao-lab.org/prediction.php). Furthermore, NCBI-CDD (https://www.ncbi.nlm.nih.gov/cdd/) and SMART (http://smart.embl.de/) were used to determine the DBD domain of potential HSF genes. The coiled-coil structure was detected by MARCOIL (https://toolkit.tuebingen.mpg.de/tools/marcoil). The NLS domain in AsHSFs was detected by cNLS Mapper (http://nls-mapper.iab.keio.ac.jp/cgi-bin/NLS_Mapper_form.cgi) [72–74]. The candidate AsHSFs lacking DBD domain or coiled-coil structure were excluded. The theoretical pI MW, GRAVY, n.c.r (%, total number of negatively charged residues (Asp + Glu)), p.c.r (%, total number of positively charged residues (Arg + Lys)), instability index (I.I.) and aliphatic index (A.I.) of HSF proteins in garlic were estimated using the Expasy proteomics server (https://web.expasy.org/computepi/). Cell-PLoc 2.0 (http://www.csbio.sjtu.edu.cn/bioinf/plant-multi/) was used to predict subcellular localization of AsHSF proteins.

### Phylogenetic relationship, gene structure and conserved motif analysis

MUSCLE [75] with default parameters was utilized to conduct multiple sequence alignment of retrieved AsHSF proteins. MEGA 7.0 was used to construct the phylogenetic tree of HSF proteins from *Allium sativum*, *Arabidopsis thaliana*, *Oryza sativa*, *Physcomitrella patens* and *Theobroma cacao* using the maximum likelihood method. The bootstrap value was 1000. The Gene Structure Display Server (GSDS: http://gsds.gao-lab.org/index.php) was utilized to elucidate the exon and intron structure of *AsHSF* genes. The conserved motifs of AsHSF proteins were analyzed by MEME (http://meme-suite.org/tools/meme)[76].

### Chromosomal localization, gene duplication, nucleotide variation and population structure of *AsHSFs*

Chromosomal locations of *AsHSF* genes were acquired from the available genome annotation information (10.6084/m9.figshare.12570947.v1). Mapchart was used to visualize chromosomal location maps. Multiple collinear scanning toolkits (MCScanX) was used to conduct gene replication events analysis of *HSF* genes with the criteria described [77]. In addition, the syntenic associations among *HSF* genes of various plants, such as *Arabidopsis thaliana*, *Oryza sativa*, *Allium sativum, Zea mays* and *Theobroma cacao*, were visualized by Dual Synteny Plotter software (https://github.com/CJChen/TBtools). The whole-genome resequencing data of 233 garlic samples were retrieved from the Genome Variation Map (accession: PRJCA006629), and detailed material information, such as geographic distribution, were showed in Table S8. The annotation of SNPs was performed using SnpEff v4.3 [78]. Furthermore, the population structure was estimated by ADMIXTURE v1.3.0 using K-values ranging from 2 to 5. Treebest v1.9.2 was used to construct the phylogenetic tree, and PCA was analyzed by the Smartpca implemented in EIGENSOFT v4.2. Vcftools v0.1.16 was used to calculate the nucleotide diversity (π) and Wright’s F-statistic (Fst).

### *Cis*-acting elements and protein interaction network of AsHSFs

The promoter region was defined as the 2000-bp region located upstream of start codon of each gene and *cis*-acting elements were identified by PlantCARE (http://bioinformatics.psb.ugent.be/webtools/plantcare/html). The protein interaction network of AsHSFs was predicted by STRING (https://cn.string-db.org/) with a confidence threshold at 0.40.

### RNA-seq data source and co-expression network analysis

A total of 185 RNA-Seq datasets were utilized to construct the co-expression network. These datasets were retrieved from the Gene Expression Omnibus (GEO) database with accession codes GSE211495, GSE186042, GSE145455, and from the Sequence Read Archive (SRA) database with accession codes PRJNA682570, PRJNA522648, PRJNA683607. Various quality parameters were used to assess the raw sequence data. Subsequently, the NGS QC Toolkit (v2.3) was employed to filter high-quality reads [79]. The filtered high-quality reads were aligned to the garlic genome using TopHat (v2.0.0) with default parameters. Then, Cufflinks (v2.0.2) was used to determine the FPKM values and read counts for each gene in the garlic. Weighted gene co-expression network analysis (WGCNA) was utilized to identify co-expressed genes with *AsHSFs* by constructing co-expression network, and co-expressed genes with the top 5% weighted values related to *AsHSFs* were selected for further investigation. To obtain the putative function of these genes, the BLAST alignment to Arabidopsis and rice proteins were implemented. Cytoscape v3.8.0 was used to visualize the co-expression networks. The GO enrichment analysis of gene sets was conducted using the clusterProfiler package in R [80].

### Plant materials and treatments

Garlic cloves (cv. Ershuizao) were planted in pots and incubated in a greenhouse. The treatment experiments were conducted after 1 month. The treatment experiments were conducted using 200 mM NaCl solution for salt stress, 4 °C for cold stress, and 30 °C for heat stress. Leaf and root samples from three plants were collected in triplicates at 0, 6, 12, 24 and 48 h after each treatment and promptly frozen using liquid nitrogen and then stored in a −80 °C freezer. Plant RNA Extraction Kits (Vazyme, Nanjing, China) was used to extract total RNA. The cDNA was synthesized by using HiScript III RT SuperMix for qPCR (Vazyme, Nanjing, China) based on the manufacturer’s manual. Real-Time PCR Detection System (LightCycler480, Roche, USA) was used to perform qRT-PCR with the following procedures: 95℃ for 3 min; 95℃ for 10 s, 60℃ for the 30 s with 40 cycles; 95℃ for 15 s; 60℃ for 1 min; 95℃ for 15 s using ChamQ Universal SYBR qPCR Master Mix (Vazyme, Nanjing, China). Primer Express (v3.0) software was used to design primer (Table S9). Relative quantification was calculated based on the 2 ^−∆∆CT^ method [81]. Three independent biological and technical replicates were performed for each PCR assay. Statistical analysis was performed by Student’s *t*-test, using SPSS software, version 17 (SPSS Inc., Chicago, IL, USA). A significant level of *P* < 0.05 was considered statistically significant.

### Yeast expression experiment and Y2H assay

To assess the high-temperature resistance of Asa6G04911 in yeast strain BY4741, *Asa6G04911* was inserted into the pYES2 vector and transformed into the BY4741 strain. One single colony was randomly selected from the positive colonies and inoculated into SD-Ura liquid medium for cultivation until reaching an optical density (OD) of 1. The initial bacterial solution concentration was uniformly adjusted to an OD of 0.8. Subsequently, dilutions were prepared at ratios of 10^− 1^, 10^− 2^ and 10^− 3^. The diluted bacterial solution was spread onto SG/-Ura plates and incubated in incubators at temperatures of 30℃ and 35℃ for a duration of 3 days.

The Y2H assays were carried out following the recommended protocol from the manufacturer (Clontech Laboratories, Inc., Palo Alto, CA, USA). The coding sequence (CDS) of *Asa6G04911* was cloned into the PGBKT7 vector as the bait, while CDS of *HSP70/90* was inserted into the PGADT7 vector as the prey. Both plasmids were introduced into yeast cells (strain AH109) through transformation. The resulting yeast transformants were cultivated on SD medium supplemented with 3-aminotriazole and X-α-gal, but deficient in Trp, Leu, His, and Ade, at 30 °C for 2–3 days.

### Electronic supplementary material

Below is the link to the electronic supplementary material.

Supplementary Material 1

Supplementary Material 2

## Data Availability

All data generated or analyzed during this study are involved in both this published article and its supplementary information files. The 185 RNA-Seq data used to construct co-expression network are publicly available from GEO under accession codes GSE211495, GSE186042, GSE145455 and SRA under accession codes PRJNA682570, PRJNA522648, PRJNA683607. The data supporting our findings can be obtained from the corresponding author upon reasonable request.
