# Supplementary material for: Genome-wide identification, classification and expression analysis of the heat shock transcription factor family in Garlic (Allium sativum L.)
Source: BMC Plant Biol. 2024 May 18;24:421. doi: 10.1186/s12870-024-05018-3 (PMC11102281; doi:10.1186/s12870-024-05018-3)
Supplement: Supplementary file 2 — Supplementary Material 2 [file 12870_2024_5018_MOESM2_ESM.docx]

**Supplementary Fig.1**

**
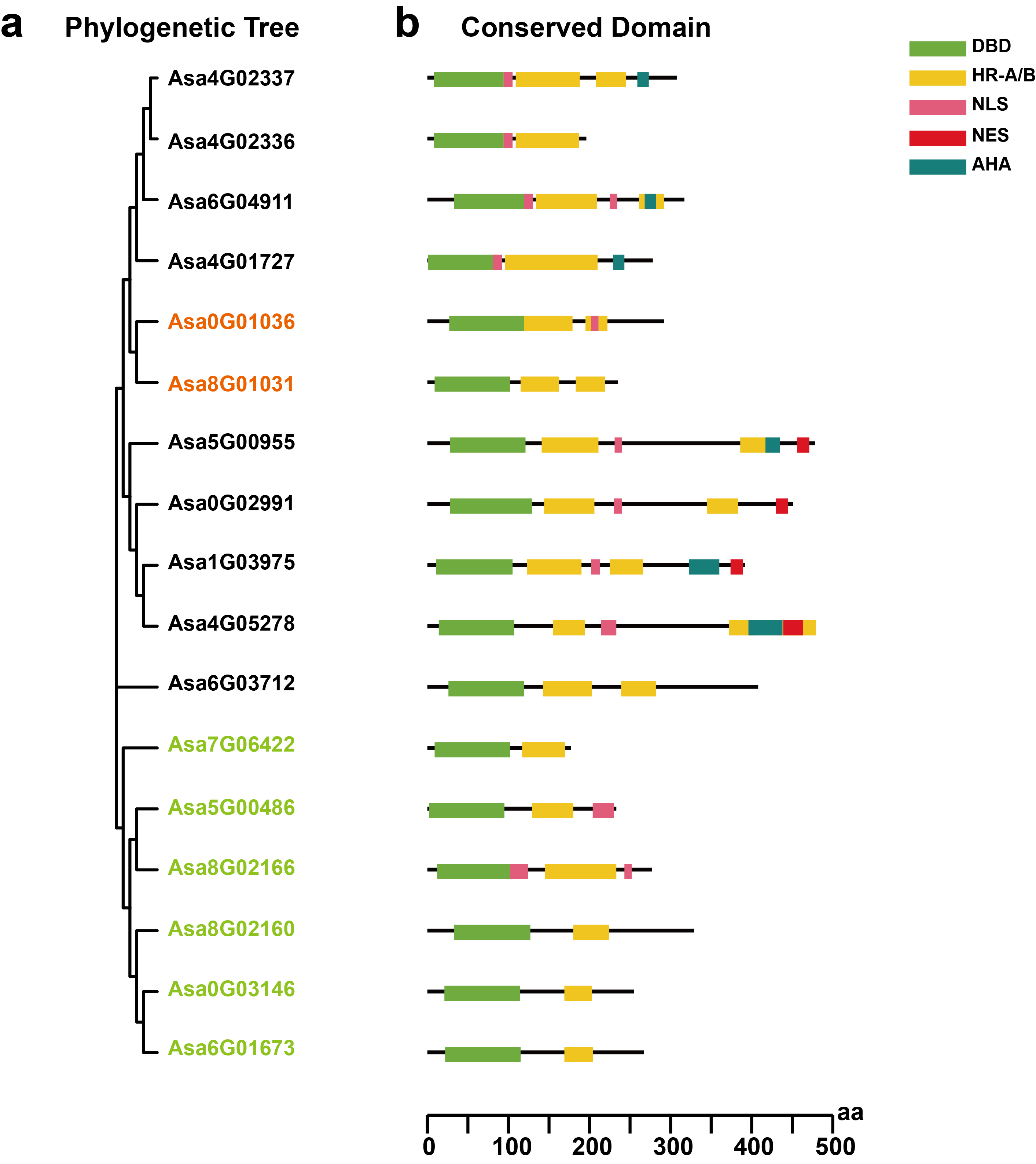
**

**Supplementary Fig.1** Phylogenetic relationships and conserved domains of AsHSF proteins. (a) The phylogenetic tree was constructed using AsHSF proteins, with members of the class A, B, and C subfamilies color-coded as black, light green, and orange, respectively. (b) The conserved domains were identified, including the DBD domain marked by green boxes, the HR-A/B domain indicated by yellow boxes, the NLS domain represented by pink boxes, the NES domain denoted by red boxes, and the AHA domain represented by cyan boxes.

**Supplementary Fig. 2**


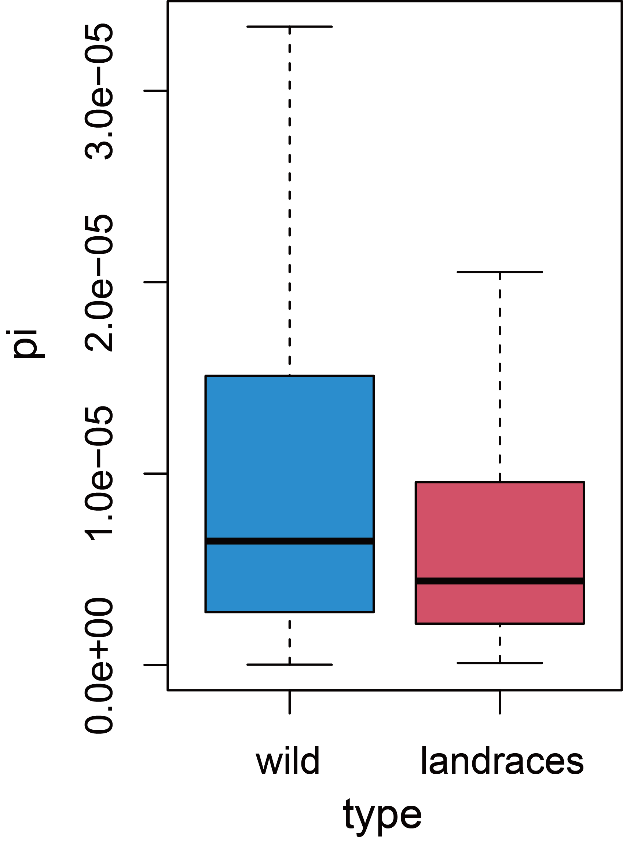


**Supplementary Fig. 2** Nucleotide diversity of wild garlic and landraces accessions. The bottom, middle, and top lines in the box represent the first, median, and third quartiles of this distribution, respectively. Nucleotide diversity was calculated according to *AsHSF*-related SNPs.

**Supplementary Fig. 3**


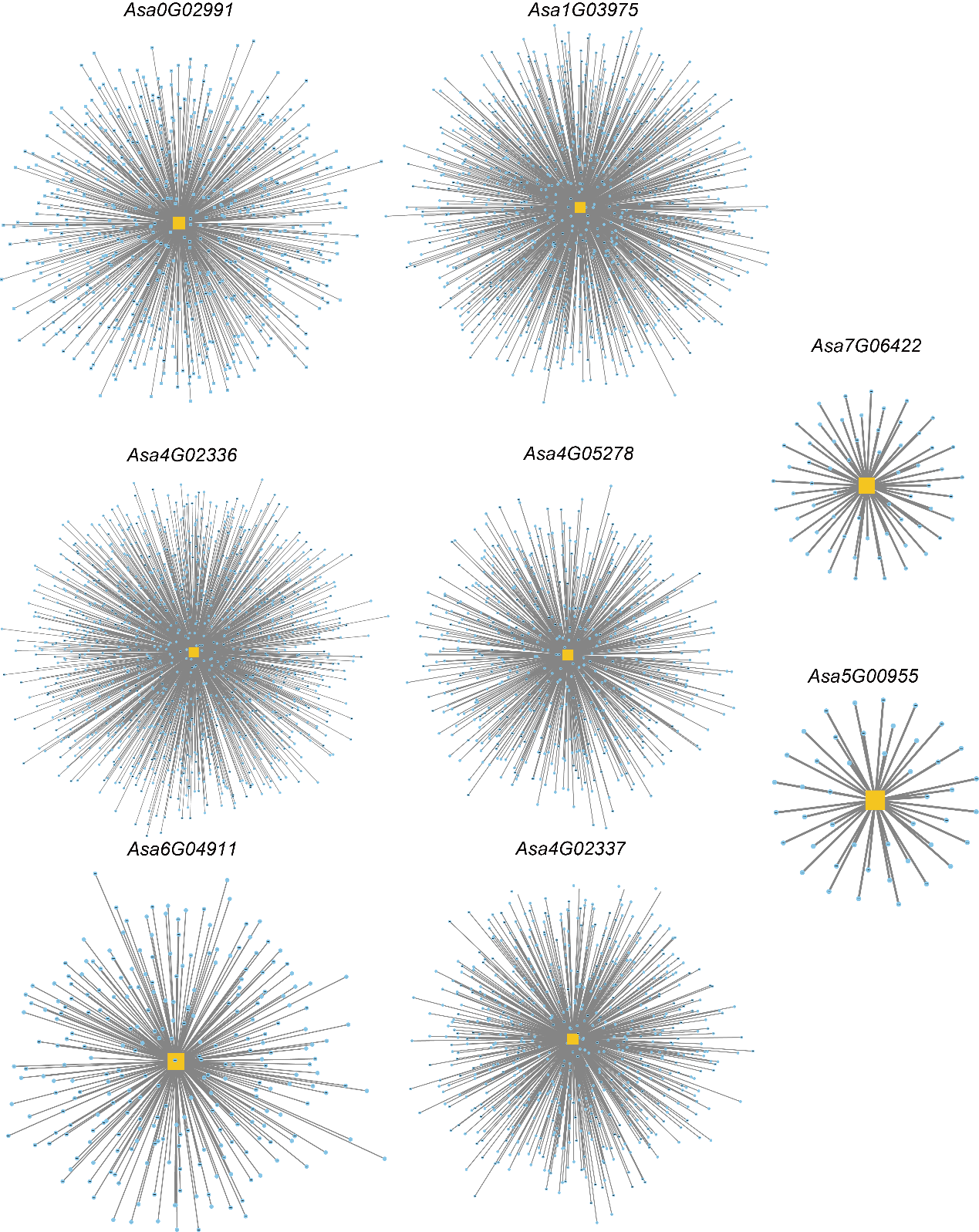


**Supplementary Fig. 3** Co-expression network analysis of eight *AsHSF* genes. Yellow rectangles indicate *AsHSF* genes, while co-expressed genes are represented by blue dots. Co-expression relationships are represented by grey lines.

**Supplementary Fig. 4**


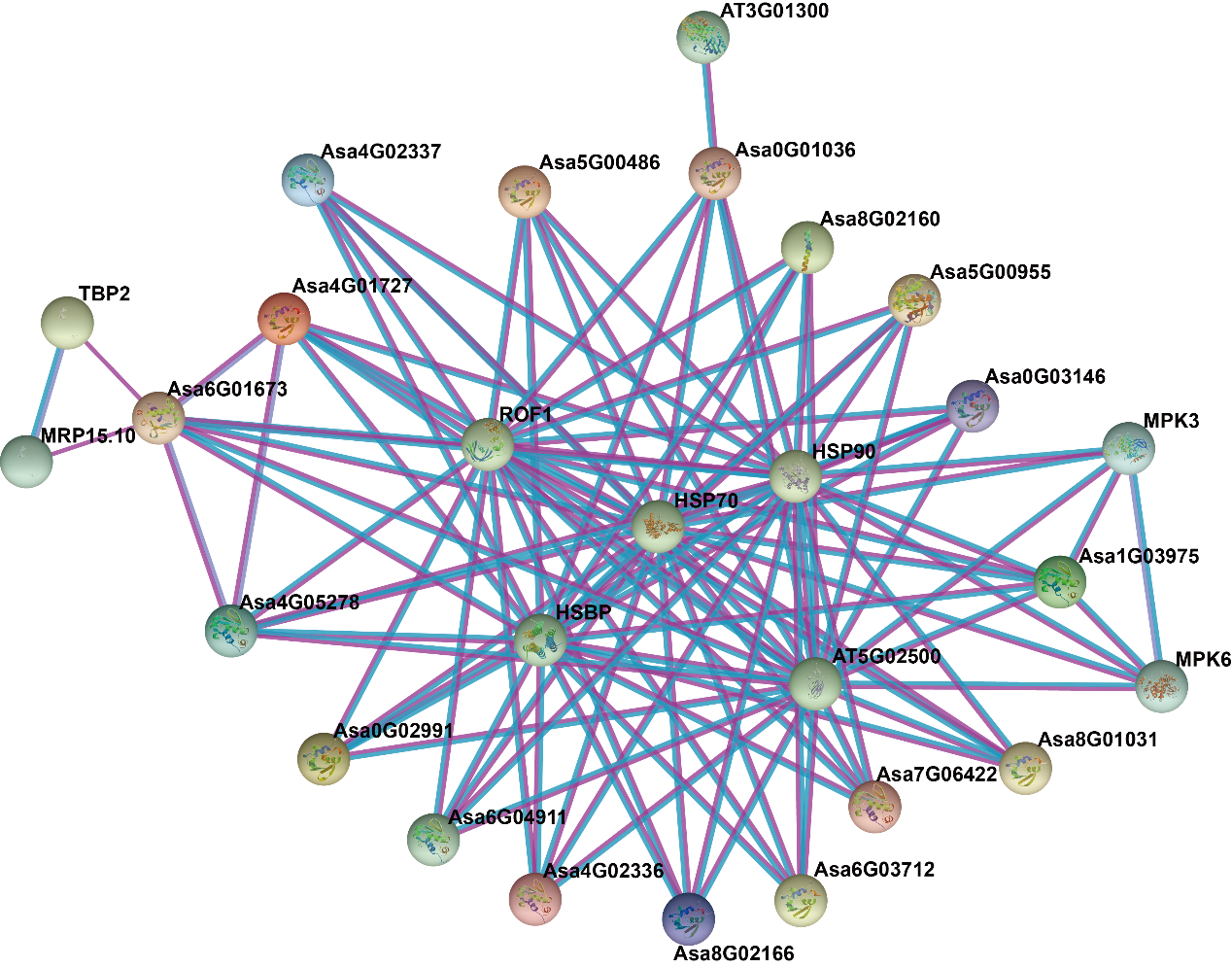


**Supplementary Fig. 4** Predicted interaction network of AsHSFs. Proteins are depicted as nodes and lines represent protein-protein interactions.

**Supplementary Fig. 5**


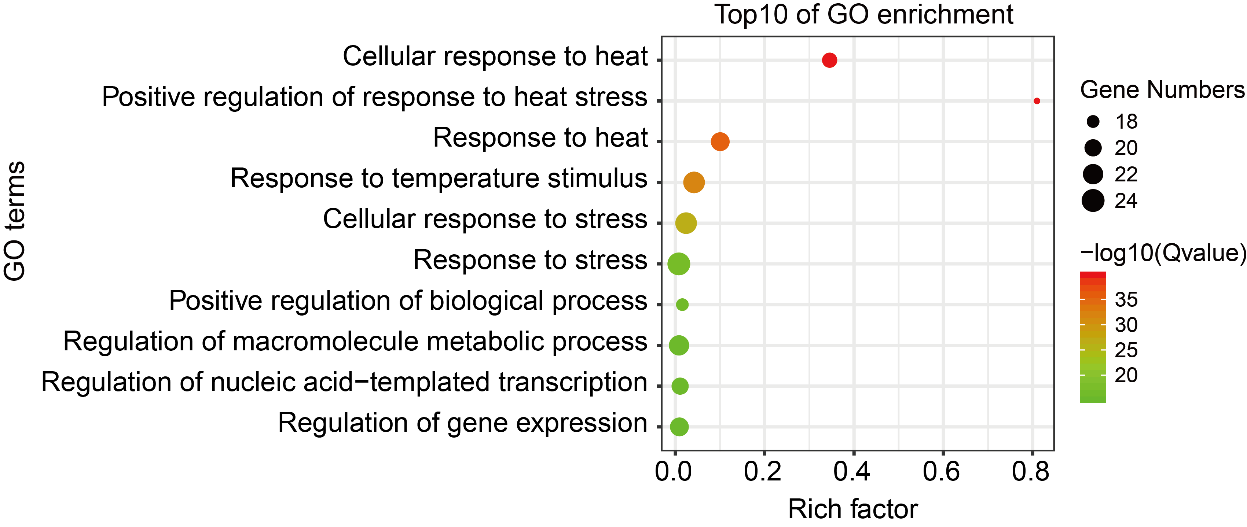


**Supplementary Fig. 5** Visualization of enriched Gene Ontology (GO) terms in the protein interaction network. Each enriched GO term is indicated as a spot, of which the size represents the number of genes enriched in particular term, and the color indicates the enrichment significance level.
